# The crosstalk between ferroptosis and mitochondrial dynamic regulatory networks

**DOI:** 10.7150/ijbs.83348

**Published:** 2023-05-21

**Authors:** Jie Li, Yu-chen Jia, Yi-xuan Ding, Jian Bai, Feng Cao, Fei Li

**Affiliations:** 1 Department of General Surgery, Xuanwu Hospital, Capital Medical University, Beijing, China.; 2 Clinical Center for Acute Pancreatitis, Capital Medical University, Beijing, China.

**Keywords:** ferroptosis, reactive oxygen species, mitochondrial homeostasis, mitochondrial fission, mitochondrial fusion, mitophagy

## Abstract

Ferroptosis is an iron-driven cell death modality characterized by iron accumulation and excessive lipid peroxidation. Ferroptosis is closely related to mitochondrial function, as indicated by studies showing that mitochondrial dysfunction and damage promote oxidative stress, which in turn induces ferroptosis. Mitochondria play crucial roles in cellular homeostasis, and abnormalities in their morphology and function are closely associated with the development of many diseases. Mitochondria are highly dynamic organelles, and their stability is maintained through a series of regulatory pathways. Mitochondrial homeostasis is dynamically regulated, mainly via key processes such as mitochondrial fission, mitochondrial fusion and mitophagy; however, mitochondrial processes are prone to dysregulation. Mitochondrial fission and fusion and mitophagy are intimately related to ferroptosis. Therefore, investigations into the dynamic regulation of mitochondrial processes during ferroptosis are important to provide a better understanding of the development of disease. In this paper, we systematically summarized changes in ferroptosis, mitochondrial fission and fusion and mitophagy to promote an in-depth understanding of the mechanism underlying ferroptosis and provide a corresponding reference for the treatment of related diseases.

## Introduction

Ferroptosis is a recently discovered mode of iron-dependent cell death that is usually accompanied by the accumulation of a large number of iron ions and excessive lipid peroxidation 1. The main morphological features are membrane shrinkage with increased membrane density and a reduced number of or loss of mitochondrial cristae 2. During ferroptosis, intracellular redox homeostasis is imbalanced, and the levels of antioxidants such as glutathione (GSH) and glutathione peroxidase 4 (GPX4) decrease, while the levels of oxidizing substances (e.g., divalent iron ions and lipid reactive oxygen species [ROS]) start to increase 3, 4. Mitochondria, as the main sources of intracellular ROS, are closely related to ferroptosis 5. Mitochondria are important organelles in cellular energy metabolism; their biological functions include the production of ATP through oxidative phosphorylation and the maintenance of redox homeostasis and calcium homeostasis 6, 7. Since the production of heme and iron-sulfur clusters occurs mainly in mitochondria, these organelles contain much mitochondrial iron and integrate iron metabolism within the cytosol. Iron binding to mitochondrial ferritin prevents ROS production, while the mutation or degradation of mitochondrial ferritin leads to mitochondrial iron overload 8, 9. Therefore, the maintenance of normal mitochondrial morphology and function is essential to ensure that cells carry out various physiological activities. Mitochondrial quality control is an important regulatory mechanism in mitochondrial homeostasis that is realized mainly through mitochondrial fusion, mitochondrial fission and mitophagy. Mitochondrial quality control processes protect mitochondria from stress-induced damage, including through the selective elimination of damaged mitochondrial proteins or dysfunctional mitochondria, thereby maintaining mitochondrial morphology, structure and function and promoting cell survival 10. Under the influence of various stress factors, mitochondria are highly susceptible to damage, which results in mitochondrial dysfunction, reduced ATP production, and increased ROS production, which have been shown to be associated with the pathology of numerous diseases. In view of the close relationship between mitochondrial homeostasis and ferroptosis in cells and the importance of this link in the development and treatment of diseases, in this review, we focus on the relationship between ferroptosis and mitochondrial function. The aim of the review is to provide a reference for better understanding this relationship and for establishing a theoretical basis for in-depth study of the relationship in the context of disease.

## Mechanisms underlying ferroptosis

**Figure 1:**
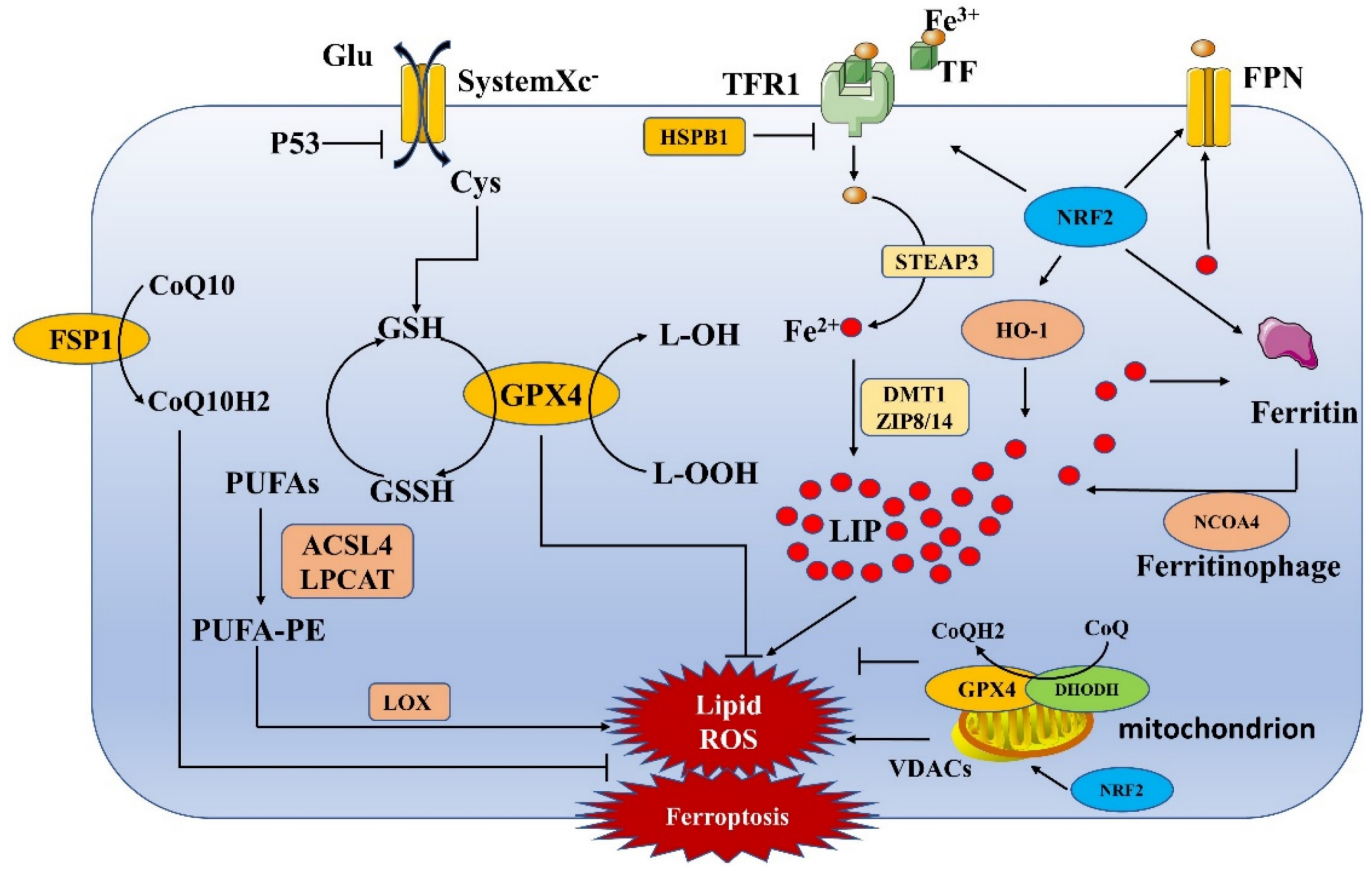
Mechanisms underlying ferroptosis. The process of ferroptosis is mainly accompanied by the accumulation of iron ions and the formation of lipid peroxides, which eventually lead to cell death. This process involves several pathways, such as the iron metabolism, System Xc-, lipid metabolism and mitochondria-related pathways.

Ferroptosis is a form of cell death induced by iron-dependent accumulation of a large number of lipid peroxides **(Figure 1)**. Both exogenous and endogenous pathways are involved in ferroptosis. The exogenous pathway induces ferroptosis mainly through the inhibition of system Xc-. The endogenous pathway network comprises various regulatory pathways, mainly lipid metabolism, iron metabolism and mitochondria-related pathways **(Table 1)** 4, 11.

### Inhibition of System Xc-induced ferroptosis

System Xc-, which consists of two subunits, SLC7A11 and SLC3A2, is a reverse transporter of amino acids and an important antioxidant complex. Cystine and glutamate are exchanged intra- and extracellularly at a 1:1 ratio through system Xc-[1]. Inhibition of system Xc- affects GSH synthesis by blocking cystine uptake, which in turn leads to a decrease in GPX4 activity. This alteration ultimately leads to a reduction in cellular antioxidant capacity and the accumulation of lipid ROS, causing the onset of oxidative damage and ferroptosis. In addition, p53 can directly inhibit System Xc-induced ferroptosis 12, 13.

### Lipid metabolism-induced ferroptosis

Ferroptosis cannot occur without lipids, and polyunsaturated fatty acids (PUFAs) are among the essential elements. Free PUFAs are esterified into membrane phospholipids and oxidized to further promote ferroptosis 14. Studies have shown that phosphatidylethanolamine (PE) containing arachidonic acid (AA) or its derivative epinephrine is the key phospholipid in ferroptosis. Acyl-CoA synthetase long-chain family member 4 (ACSL4) is important for the synthesis of phospholipids, while lysophosphatidylcholine acyltransferase 3 (LPCAT3) is important for the insertion of PUFAs into membrane phospholipids 15. ACSL4 and LPCAT3 together influence lipid peroxidation and are key nodes in the regulation of ferroptosis. Ultimately, PUFA-PEs contribute to further oxidation catalyzed by lipoxygenase (LOX), eventually inducing ferroptosis 16. Nuclear factor erythroid 2-related factor 2 (NRF2) can also regulate lipid metabolism, for example, through the ligand-mediated regulation of the transcription factor peroxisome proliferator-activated receptor γ (PPARγ)^17^.

### Iron metabolism-regulated ferroptosis

Ferroptosis is primarily driven by iron-dependent lipid peroxidation. Many aspects of iron metabolism, such as iron uptake, storage and utilization, play important roles in the regulation of ferroptosis. Nonheme iron (Fe^3+^) binds to transferrin (TF), and then TF receptor protein 1 (TFR1) mediates the uptake of the TF-Fe^3+^ complex into the cell. Fe^3+^ is reduced to Fe^2+^ by six-transmembrane epithelial antigen of prostate 3 (STEAP3) and is then stored by divalent metal transporter 1 (DMT1) or zinc-iron regulatory protein family 8/14 (ZIP8/14) as part of the cytoplasmic labile iron pool (LIP) or as ferritin; the excess Fe^2+^ is oxidized to become Fe^3+^ outside the cell by the membrane iron transport export protein (Fpn)^18^. Heme oxygenase-1 (HO-1) accelerates ferroptosis by increasing the number of iron ions, while heat shock protein B1 (HSPB1) inhibits ferroptosis by reducing iron ion uptake via inhibition of TRF1 expression 19, 20. In addition, heme degradation and nuclear receptor coactivator 4 (NCOA4)-mediated ferritinophagy increase the size of the LIP, sensitizing cells to ferroptosis via the Fenton reaction 21, 22. In addition, the metabolism, storage, and transport of many iron-related proteins, including ferritin, TFR, Fpn, and HO-1, are regulated by NRF2. NRF2 regulation thus alters intracellular iron levels, which in turn affects ferroptosis in cells 23.

### Regulation of ferroptosis with mitochondrial involvement

Mitochondria are the main sources of cellular ROS, and mitochondrial ROS production may contribute to ferroptosis by promoting lipid peroxidation 24. Mitochondria are also the main organelles for ATP production, and electron transport and proton pumping in mitochondria play important roles in the induction of ferroptosis. When ATP is deficient, the energy sensor AMP-activated protein kinase (AMPK) is activated, which affects the activity of acetyl-CoA carboxylase (the rate-limiting enzyme for fatty acid synthesis) and inhibits ferroptosis 25, 26. Mitochondria also have additional functions in biosynthesis and cellular metabolism that are mediated by the TCA cycle and various anaplerotic reactions that complement the TCA cycle 27. The potential mechanism by which the TCA cycle regulates ferroptosis may be related to the function of this cycle in electron transport and fatty acid biosynthesis 5.

GPX4 and dihydroorotate dehydrogenase (DHODH) in mitochondria are key members of the two systems that protect cells from ferroptosis. Disabling one system forces cells to become more dependent on the other, while simultaneous inhibition of mitochondrial GPX4 and DHODH triggers ferroptosis mainly via mitochondrial lipid peroxidation 5, 28. Mechanistically, DHODH acts in parallel with mitochondrial GPX4 (independent of cytoplasmic GPX4 or FSP1) to inhibit ferroptosis in the mitochondrial inner membrane (MIM) by reducing CoQ to CoQH2 (a radical-trapping antioxidant with antiferroptotic activity) 28.

The antioxidant transcription factor NRF2 is also an important regulator of mitochondrial function and has important effects on ferroptosis. NRF2 can bind to mitochondria and thus can indicate and influence changes in mitochondrial function 29. Knockdown of NRF2 in mice impairs mitochondrial function, while activation of NRF2 enhances mitochondrial function and enhances stress resistance 30, 31. NRF2 regulates mitochondrial function in multiple ways and affects the dynamic homeostasis of mitochondria. NRF2 can affect mitochondrial biogenesis by regulating the expression of PGC-1α, NResF1, NResF2, TFAM and mitochondrial genes 32; it can also regulate mitophagy through a P62 dependent, PTEN-induced kinase 1 (PINK1)/Parkin-independent mechanism 33. Furthermore, NRF2 can regulate proteasomal genes and MFN2, thereby regulating mitochondrial fission and mitochondrial fusion, respectively 34. Overall, NRF2 regulates mitochondrial function, and alterations in mitochondrial function further influence ferroptosis.

### Other mechanisms

Other pathways, such as the mitochondrial voltage-dependent anion channel pathway, p53 pathway, FSP1-COQ10-NAD(P)H pathway, NRF2-regulated iron and lipid metabolism pathways and sulfur transfer pathway, can also influence the course of ferroptosis 13, 17, 23, 35-38. Abnormalities in these pathways directly or indirectly lead to an imbalance in the intracellular oxidative response, contributing to ferroptosis.

## Dynamic regulation of mitochondria

Mitochondria are constantly undergoing dynamic changes within a cell. Mitochondrial self-regulation occurs through constant fission and fusion, providing energy for cellular homeostasis and regulating macroautophagy, calcium homeostasis, innate immunity, signaling and apoptosis. The relative balance of mitochondrial fission and fusion is essential to maintain the quality and function of mitochondria and is an important basis for normal cellular activity 7, 39. In contrast, mitophagy involves the sequestration and removal of damaged mitochondria from a cell through a specific autophagic pathway, thereby maintaining intracellular mitochondrial homeostasis 40. The interaction and coregulation of mitophagy and mitochondrial fission and fusion are mechanisms important for the maintenance of mitochondrial homeostasis and mitochondrial quality **(Table 2)** 41, 42.

### Mitochondrial fission

**Figure 2:**
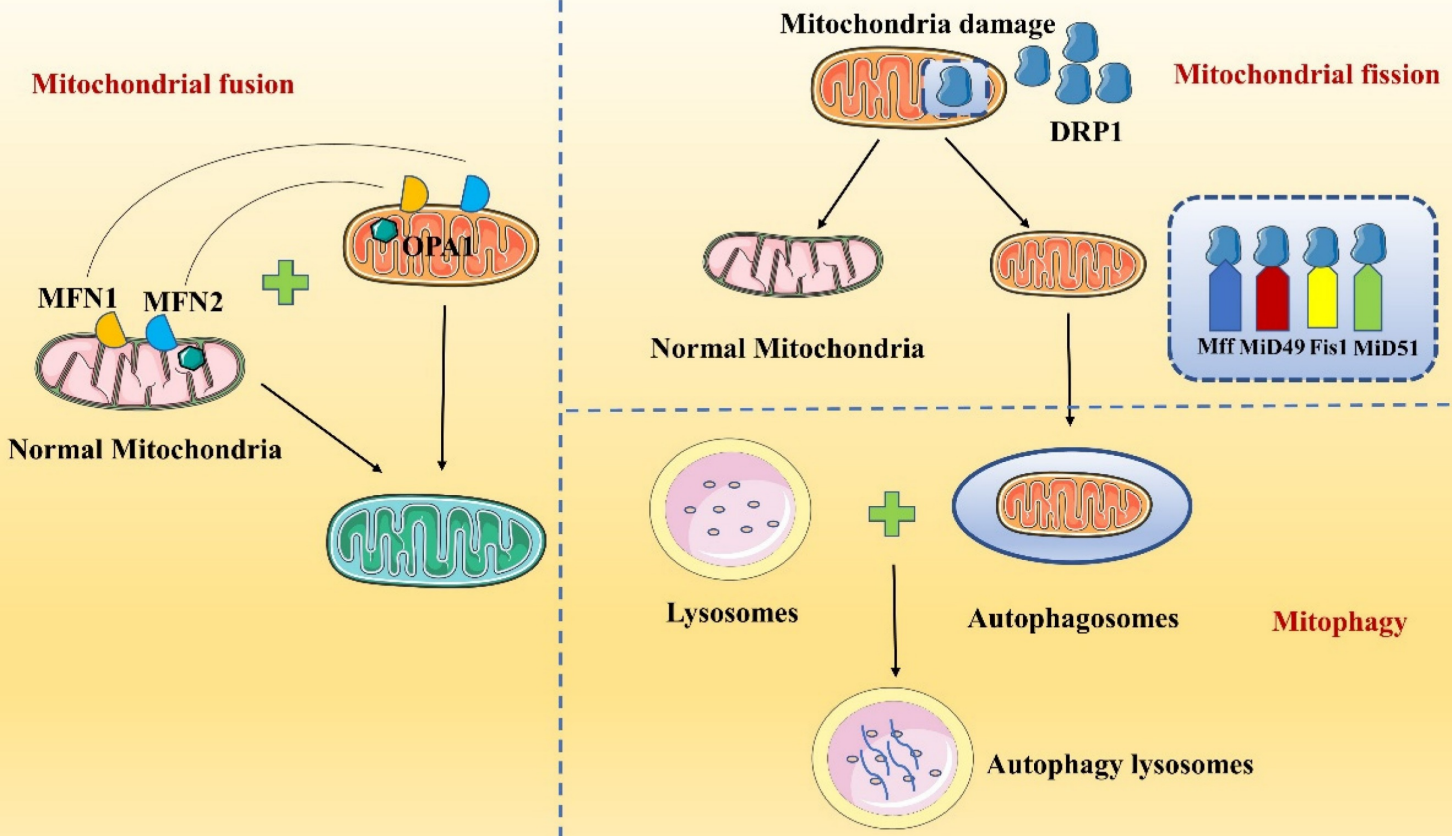
Regulation of mitochondrial homeostasis. Mitochondrial homeostasis is maintained mainly by mitochondrial fission, mitochondrial fusion and mitophagy.

Mitochondria are double-membrane organelles with a mitochondrial outer membrane (MOM) and a MIM, which are separated by the intermembrane space (IMS). Mitochondrial fission contributes to quality control because it causes damaged mitochondria to split into two mitochondria: one mitochondrion with normal function and one with abnormal function **(Figure 2)**. Then, abnormally functioning mitochondria are eliminated by mitophagy to maintain mitochondrial homeostasis *in vivo* 43. During mitochondrial fission, GTPase dynamin-related protein 1 (DRP1), also known as dynamin-1-like protein (DNM1L), is a key mediating factor 44-47. DRP1 interacts with a variety of protein receptors in the MIM or MOM; these receptors include mitochondrial fission factor (Mff), mitochondrial fission protein 1 (Fis1), mitochondrial dynamics protein 49 (MiD49) and mitochondrial dynamics protein 51 (MiD51) 48. Multiple DRP1 molecules are recruited to a single mitochondrion and bind to receptors, closely surrounding the mitochondrion to form a ring finger structure. Then, via their GTPase activity, DRP1 molecules hydrolyze GTP, which leads to MIM and MOM permeabilization and causes mitochondrial fission. After mitochondrial fission, DRP1 is relocalized to the cytosol 46, 47. Notably, DRP1 action is regulated by posttranslational modifications, such as ubiquitination and phosphorylation, and by metabolic signaling 49.

### Mitochondrial fusion

Mitochondrial fusion requires both the MOM and MIM. Fusion involves multiple proteins and is initiated by the tethering of mitochondria followed by sequential fusion of the MOMs and MIMs **(Figure 2)**^50^. Three main proteins participate in mitochondrial fusion in mammalian cells: mitochondrial fusion protein 1 (MFN1), mitochondrial fusion protein 2 (MFN2) and optic atrophy 1 protein (OPA1) 51, 52. MOM fusion is mediated primarily by MFN1 and MFN2, while MIM fusion is mediated by OPA1. MFN1 and MFN2 are in the MOM and form homodimeric or heterodimeric structures to connect two adjacent mitochondria, contributing to mitochondrial transmembrane protein interactions 53, 54. Next, the MFN GTPase structural domain hydrolyzes GTP, causing membrane conformational changes that initiate mitochondrial fusion. Hence, MFN1 is considered the major GTP-dependent membrane-bound protein for mitochondrial fusion; mitochondria cannot fuse in cells lacking MFN1, while MFN2 is thought to act at a later stage of the fusion process 55. MOM fusion is followed by MIM fusion, which is mediated mainly by OPA1, a kinesin-related GTP enzyme with two isoforms, long-OPA1 (L-OPA1) and short-OPA1 (S-OPA1), that synergistically regulate mitochondrial fusion. When the transmembrane potential of the MIMs is intact, the L-OPA1 isoform mediates mitochondrial fusion by binding to cardiolipin (CL)^52^. When the MIM potential is lost, such as in a stress state, L-OPA1 is rapidly hydrolyzed to S-OPA1, leading to mitochondrial fusion. During mitochondrial fusion, information and material are transferred between the MIMs and MOMs. In summary, MFN1 may interact with the MIM protein OPA1, and OPA1 induces MIM fusion by relying on MFN1 but not on MFN2^56^.

### Mitophagy

#### PINK1/Parkin and PINK1/Parkin-independent pathways

**Figure 3:**
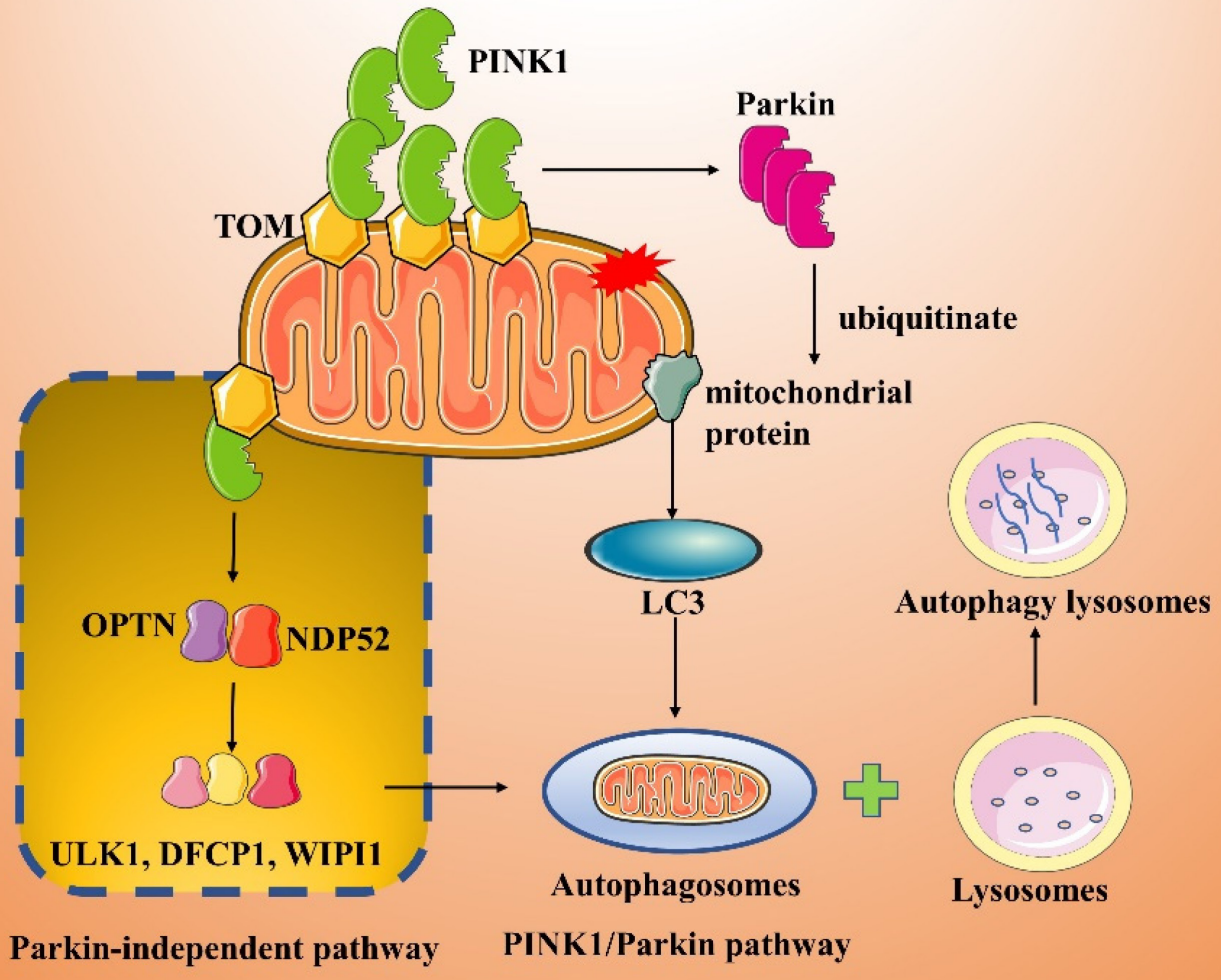
Parkin-dependent and -independent pathways of mitophagy activated by PINK1.

The PINK1/Parkin pathway is the classical and most extensively studied pathway in mitophagy **(Figure 3)**^57^-^59^. PINK1 is a mitochondrial serine/threonine kinase containing a mitochondrial targeting sequence (MTS), while Parkin is an E3 ubiquitin ligase present in the cytosol. Both proteins play important roles in mitochondrial autophagic degradation 60. Under normal cellular physiological conditions, PINK1 is sequentially translocated into the MOM by translocases. The MTS sequence of PINK1 is then excised, leading to its degradation. When a mitochondrion is damaged, its membrane potential rapidly decreases, and the import of PINK1 into the mitochondrion is inhibited; therefore, PINK1 accumulates on the MOM 60. The accumulated PINK1 recruits and activates Parkin, which in turn polyubiquitinates a variety of mitochondrial protein substrates 61, 62. Ultimately, in the presence of LC3 splice proteins, autophagosomes are targeted to the mitochondrion, inducing mitophagy.

Lazarou et al. 63 showed that PINK1 directly recruits optineurin (OPTN) and NDP52 to damaged mitochondria in a Parkin-independent manner and subsequently recruits and activates the autophagy initiator unc-51-like kinase 1 (ULK1), double FYVE-containing protein 1 (DFCP1) and WD repeat domain phosphoinositide interacting 1 (WIPI1), which in turn induce mitophagy **(Figure 3)**. In addition, synphilin-1 overexpression mediates the accumulation of large amounts of PINK1 on the MOM, and the PINK1-synphilin-1 complex induces mitophagy by recruiting SIAH-1 to accelerate the ubiquitination of proteins in damaged mitochondria 64.

#### Receptor-mediated pathways

**Figure 4:**
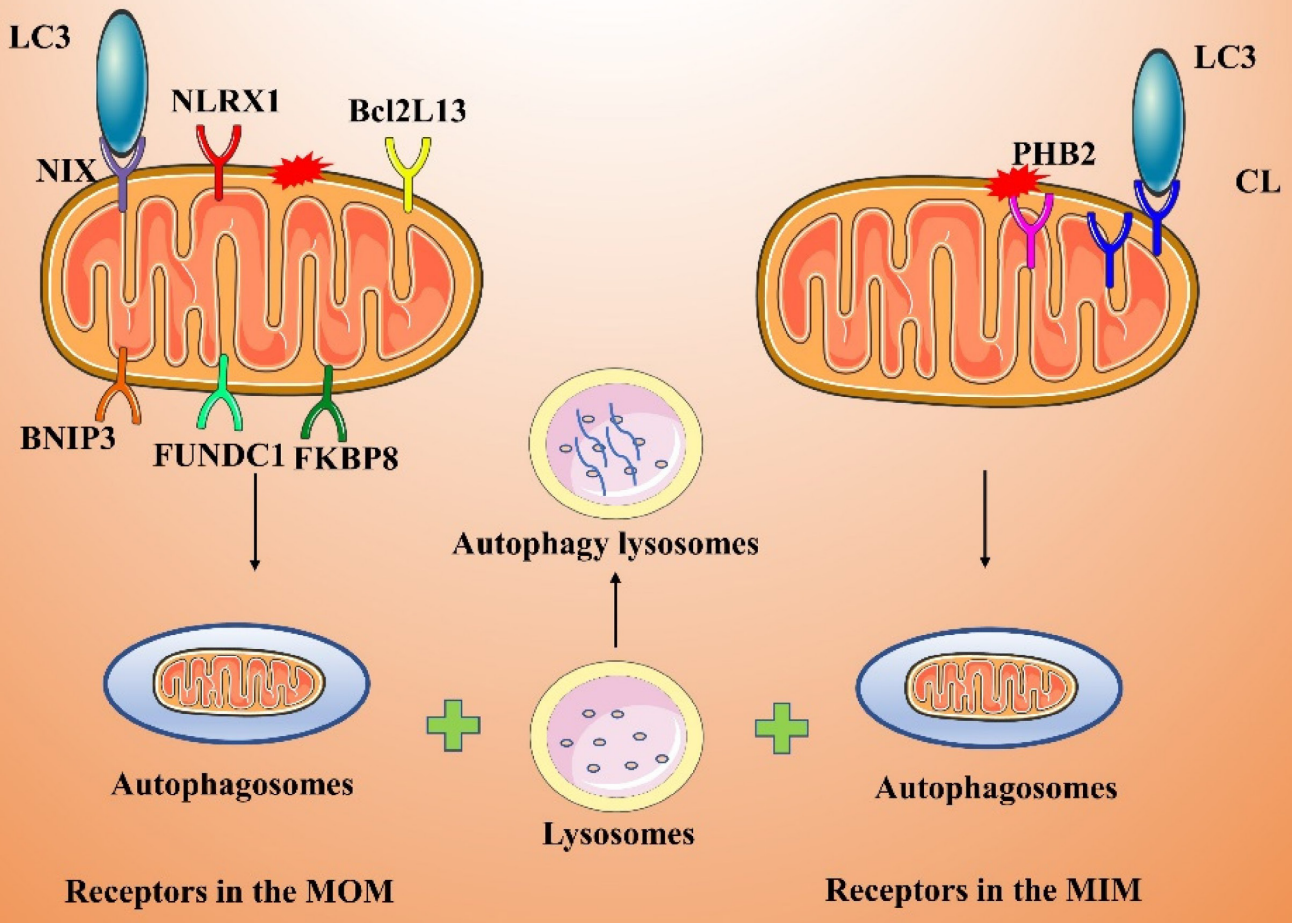
Receptor-mediated activation pathway of mitophagy.

Some mitochondrial membrane proteins are LC3 receptors that directly recognize and bind LC3, thereby inducing mitophagy. LC3 receptors include Nip3-like protein X (NIX/BNIP3L), BNIP3, FUNDC1, NLRX1, FKBP8, Bcl2L13, PHB2, and CL **(Figure 4)**.

##### Receptors in the MOM

NIX and BNIP3 are in the MOM, and both contain a BH3 structural domain that directly binds LC3 to induce mitophagy 65, 66. These two receptors mediate mitophagy primarily under hypoxic or postinjury conditions; in particular, hypoxia leads to effective inhibition of mitophagy by inhibiting the actions of NIX and BNIP3. Under hypoxic conditions, intracellular ROS production increases, and hypoxia-inducible factor 1 (HIF-1) can bind to NIX/BNIP3, thereby increasing the expression level of NIX/BNIP3 and promoting mitophagy 67.

FUNDC1 is an MOM protein that, under hypoxic conditions, induces mitophagy by binding directly to LC3^68^, ^69^. FUNDC1 is in a phosphorylated form in the physiological state, but under hypoxia, FUNDC1 undergoes dephosphorylation, which enhances its interaction with LC3 and promotes mitophagy 70, 71. In addition, FUNDC1-induced mitophagy activity is regulated by ubiquitination. Under hypoxic conditions, membrane-associated ring finger protein 5 (MARCH5) promotes the ubiquitination of FUNDC1 at Lys119, which leads to the degradation of FUNDC1. In contrast, knocking down MARCH5 impairs the ubiquitination and degradation of FUNDC1, thereby enhancing hypoxia-induced mitophagy 72.

NLRX1 is an important cytoplasmic pattern recognition receptor and a member of the nucleotide-binding oligomerization domain (NOD)-like receptor (NLR) family. NLRX1 has been identified as a novel mitophagy-related receptor 73, 74. For example, NLRX1 is essential for the induction of mitophagy by *Listeria monocytogenes*. Specifically, the virulence factor LLO in *L. monocytogenes* induces the oligomerization of NLRX1, which in turn promotes the intercalation of the LC3-interacting region (LIR) of NLRX1 into LC3 to induce mitophagy, maintain cellular homeostasis, and promote the survival of *L. monocytogenes* 73*.*

FKBP8 is in the MOM and interacts directly with LC3A to induce mitophagy in a Parkin-independent manner 75, 76. When damaged mitochondria are engulfed by autophagosomes, FKBP8 is translocated from mitochondria to the endoplasmic reticulum (ER) to prevent its own degradation.

BCL2L13 is a homolog of yeast Atg32 and is known to induce mitophagy in cells lacking Atg32. BCL2L13 localizes to the MOM and, similar to other LC3 receptors, binds directly to LC3 through its LIR to induce mitophagy 77. The ability of BCL2L13 to induce mitophagy is regulated by its phosphorylation. Phosphorylation of BCL2L13 Ser272 promotes BCL2L13 binding to LC3 and enhances its mitophagy-inducing action 78.

##### Receptors in the MIM

PHB2 and CL both localize to the MIM 79, 80. Parkin-mediated ubiquitination-related degradation of MOM proteins leads to MOM damage, resulting in exposure of the MIM protein PHB2, which in turn binds to LC3. Moreover, PHB2 induces mitophagy by stabilizing PINK1 and promoting the recruitment of PRKN/Parkin, which ubiquitinates the PHB2 protein, and by recruiting OPTN to mitochondria 81. Ultimately, autophagosomes are compensatorily recognized by different receptor proteins localized to the MOM and MIM of damaged mitochondria, perhaps improving the efficiency of mitophagy.

CL is a membrane lipid and LC3 receptor on the MIM that induces mitophagy 80, 82. CL, a characteristic phospholipid of the MIM, is involved in lipid-protein interactions and is a raw material necessary for mitochondrial functions (e.g., cristae formation and membrane fusion). After treating cells with CCCP or the mitophagy inducer rotenone, Chu et al. observed that CL was transferred from the MIM to the MOM and was highly expressed on the MOM 80. Inhibiting molecular interactions between CL and proteins, interfering with CL synthase or restricting CL transport to the MOM blocks mitophagy.

## Ferroptosis and the dynamic regulation of mitochondria

Mitochondria are essential for the process of ferroptosis. Mitochondria are semiautonomous organelles that play key roles in the cellular stress response, energy metabolism and cell survival. Mitochondrial quality control is accomplished primarily through the dynamic regulation of mitochondrial fission, mitochondrial fusion and mitophagy. Maintaining mitochondrial quality control is important for mitochondrial homeostasis *in vivo*. Pathological conditions, such as mtDNA mutations, mitochondrial fusion and fission imbalance, and dysregulated mitophagy, can lead to abnormal mitochondrial morphology, oxidative phosphorylation dysfunction, reduced ATP synthesis, cytochrome C release, and activation of mitochondria-dependent cell signaling, which in turn can cause mitochondrial dysfunction. Changes in mitochondrial dynamics regulation can influence mitochondrial function and thus regulate the development of ferroptosis.

### Ferroptosis and mitochondrial fission

**Figure 5:**
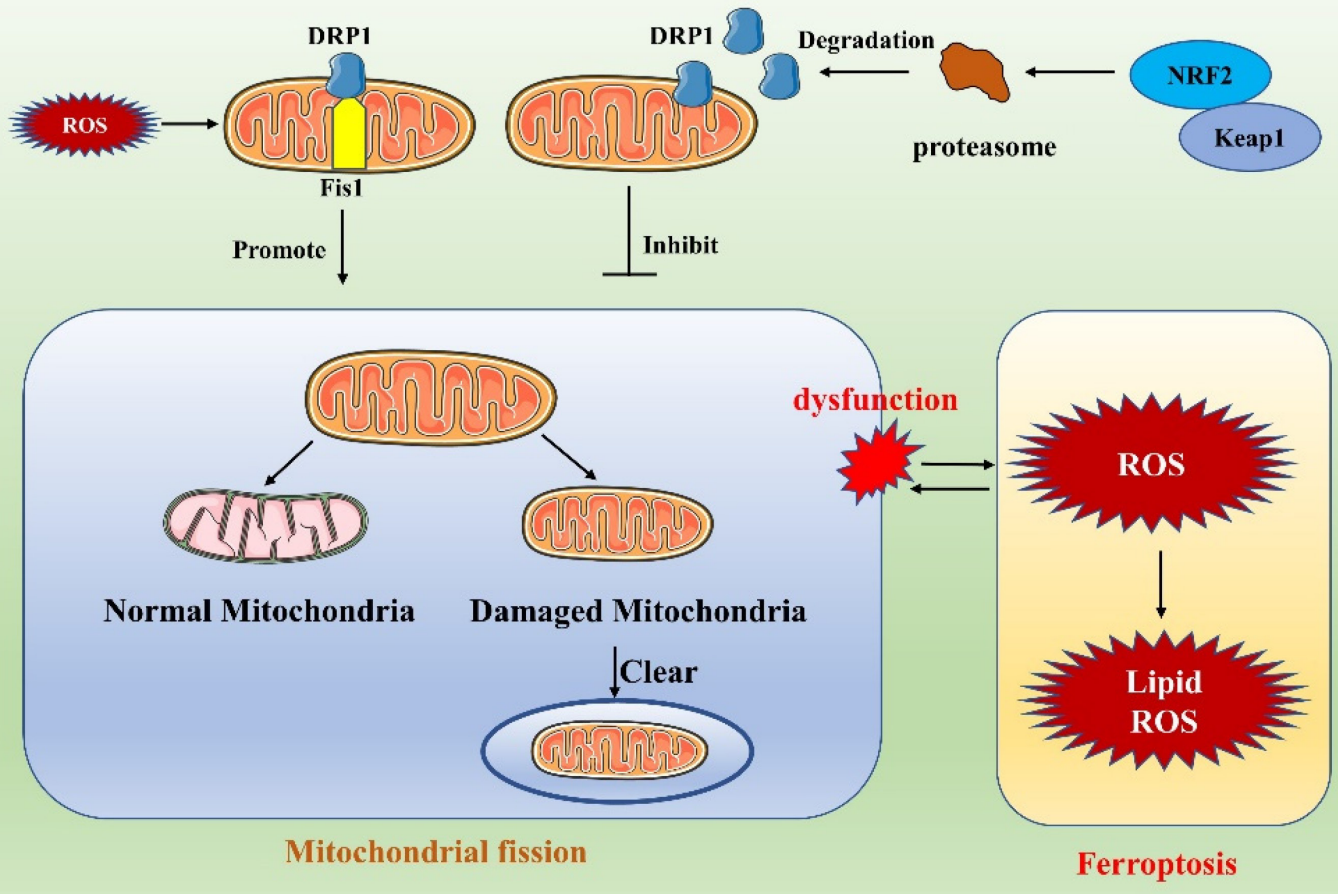
Link between ferroptosis and mitochondrial fission.

Ferroptosis, which is characterized by iron dependence and lipid peroxide accumulation, is a regulated cell death modality that depends on ROS 4. Mitochondrial fission is an important means of cellular self-regulation and plays a key role in maintaining mitochondrial function. Ferroptosis and mitochondrial fission intersect **(Figure 5)**. For example, NRF2, an important factor in the regulation of ferroptosis, regulates proteasomal genes that contribute to the degradation of DRP1, thereby inhibiting mitochondrial fission 83. The interaction of mitochondrial fission with ferroptosis is complex and diverse. Liu et al. 84 found that cisplatin-induced intestinal injury involves ferroptosis and that the protein and mRNA expression levels of the mitochondrial fission-related proteins DRP1 and Fis1 are increased during ferroptosis in injured intestines. They found that vitamin D3 treatment alleviates the accumulation of ROS by inhibiting ROS production, thereby attenuating excessive mitochondrial fission and increasing mitochondrial ATPase activity. This treatment also increases cellular antioxidant capacity, inhibits the accumulation of ROS and malondialdehyde, and suppresses ferroptosis. These outcomes strongly suggest an interaction between ferroptosis and mitochondrial fission 84. Other recent studies have shown that the relationship between mitochondrial fission and ferroptosis is profoundly involved in the development of cancer. Notably, Liu et al. found that the ferroptosis inducer erastin sensitizes non-small cell lung cancer (NSCLC) cells to celastrol by activating the ROS-mitochondrial fission-mitochondrial autophagy pathway, enhancing the anticancer effects of celastrol 85. Malignant mesothelioma (MM) is an aggressive tumor with a poor prognosis. Mitochondrial fission is enhanced after inhibition of carbonic anhydrase 9, a membrane-associated α-CA that has been targeted by drugs in a variety of cancers, and ferroptosis has been shown to participate in this process 86. Similarly, when ZENG et al. inhibited SHARPIN gene expression in cholangiocarcinoma, they found that ROS accumulation and mitochondrial fission were suppressed and that ferroptosis in cells was promoted through the p53/SLC7A11/GPX4 signaling pathway 87. These findings illustrate that in the treatment of tumors, targeting of a single gene may regulate mitochondrial fission and ferroptosis simultaneously, enhancing the anticancer effect. This may provide new ideas not only for drug development but also for a new level of tumor treatment.

### Ferroptosis and mitochondrial fusion

**Figure 6:**
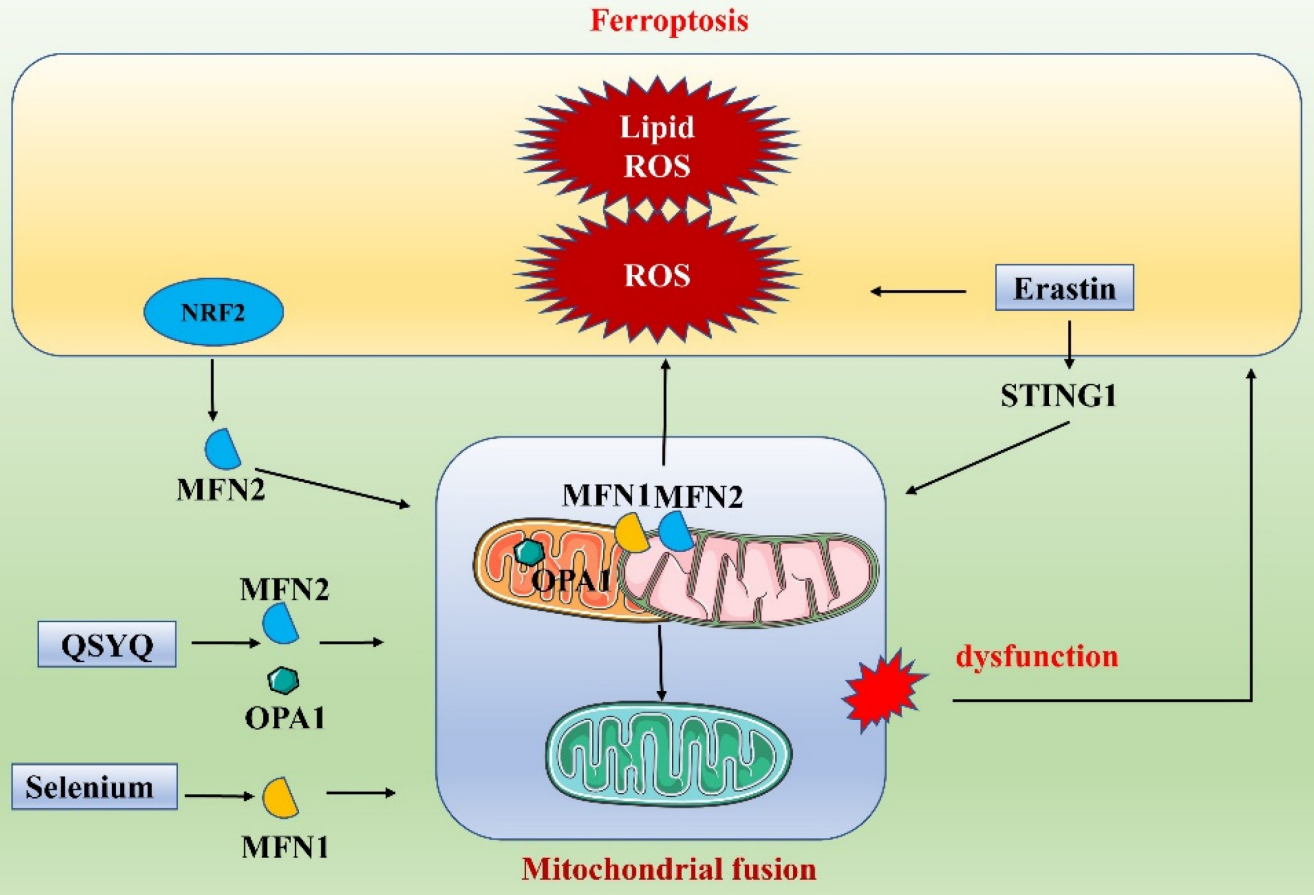
Link between ferroptosis and mitochondrial fusion.

Under normal conditions, mitochondria are altered as fission and fusion processes are balanced. However, when this dynamic balance is disrupted, mitochondria may become functionally altered or defective. NRF2, a key factor in ferroptosis, also exerts an important regulatory function in mitochondrial fusion. NRF2 activation can promote the expression of MFN2, further promoting mitochondrial fusion. This suggests a close link between mitochondrial fusion and ferroptosis and a network of coregulated gene interactions 88. In recent years, many researchers have gradually explored the specific processes of ferroptosis and mitochondrial fusion **(Figure 6)**. Li et al. found that targeting mitochondrial fusion-mediated ferroptosis may be a promising strategy for future cancer therapy 89. Specifically, they found that the ER protein STING1 (also known as STING or TMEM173) promoted ferroptosis in human pancreatic cancer cell lines by increasing mitochondrial fusion protein MFN1/2-dependent mitochondrial fusion. The classical ferroptosis inducer erastin induced STING1 translocation from the ER to mitochondria, where it accumulated, and the accumulated STING1 bound to MFN1/2, triggering mitochondrial fusion. The increases in ROS and fatty acid levels during mitochondrial fusion led to subsequent ROS production and lipid peroxidation, promoting ferroptosis. In contrast, deletion of the STING1 or MFN1/2 genes reduced the sensitivity of pancreatic cancer cells to ferroptosis. In addition, in a study on cerebral ischemia/reperfusion (I/R) injury, Shi et al. elaborated on the link between mitochondrial fusion and ferroptosis 90. They found that treatment with selenium attenuated cerebral I/R injury and increased mouse survival. Furthermore, in a mouse model of middle cerebral artery occlusion (MCAO) and in N2a cells subjected to oxygen/glucose deprivation/reoxygenation, selenium treatment significantly attenuated oxidative stress and iron ion accumulation. Mechanistically, selenium promoted mitochondrial fusion through upregulation of MFN1 expression, which in turn attenuated oxidative stress and ferroptosis. An increasing number of drugs have been found to regulate both mitochondrial fusion and ferroptosis. For example, QiShenYiQi dripping pill (QSYQ) reduces myocardial ischemia-induced mitochondrial prolapse by improving mitochondrial homeostasis and biosynthesis. Specifically, QSYQ promotes mitochondrial biogenesis (PGC-1α, Nrf1 and TFAM) and mitochondrial fusion (MFN-2 and OPA1) and inhibits excessive mitochondrial fission (phosphorylation of DRP1 at Ser616) 91. These results suggest that dysfunction of mitochondrial fusion, which is part of the dynamic regulation of mitochondria, affects overall mitochondrial function and consequently affects ferroptosis. In conclusion, dysregulation of mitochondrial fusion leads to an energy crisis, coordinates ferroptosis, and affects the redox state of the cell.

### Ferroptosis and mitophagy

Macroautophagy (hereafter referred to as autophagy) is a conserved cellular process capable of maintaining homeostasis *in vivo* by degrading various biomolecules and organelles via the lysosomal pathway 92. Recent studies have progressively revealed that there is an important interaction between ferroptosis and autophagy: ferroptosis requires the autophagic mechanism for its execution 93, 94. Among the discovered autophagic processes, selective types of autophagy (e.g., ferritinophagy, lipophagy, mitophagy, clockophagy, etc.) play important roles in driving cells toward ferroptosis. The specific processes include NCOA4-promoted ferritinophagy, BECN1-mediated systemic Xc- inhibition, RAB7A-dependent lipophagy, STAT3-induced lysosomal membrane permeabilization, SQSTM1-dependent clockophagy, HSP90-associated chaperone protein-mediated autophagy and PINK1-associated mitophagy, all of which regulate the cellular ferroptosis process 93, 94.

**Figure 7:**
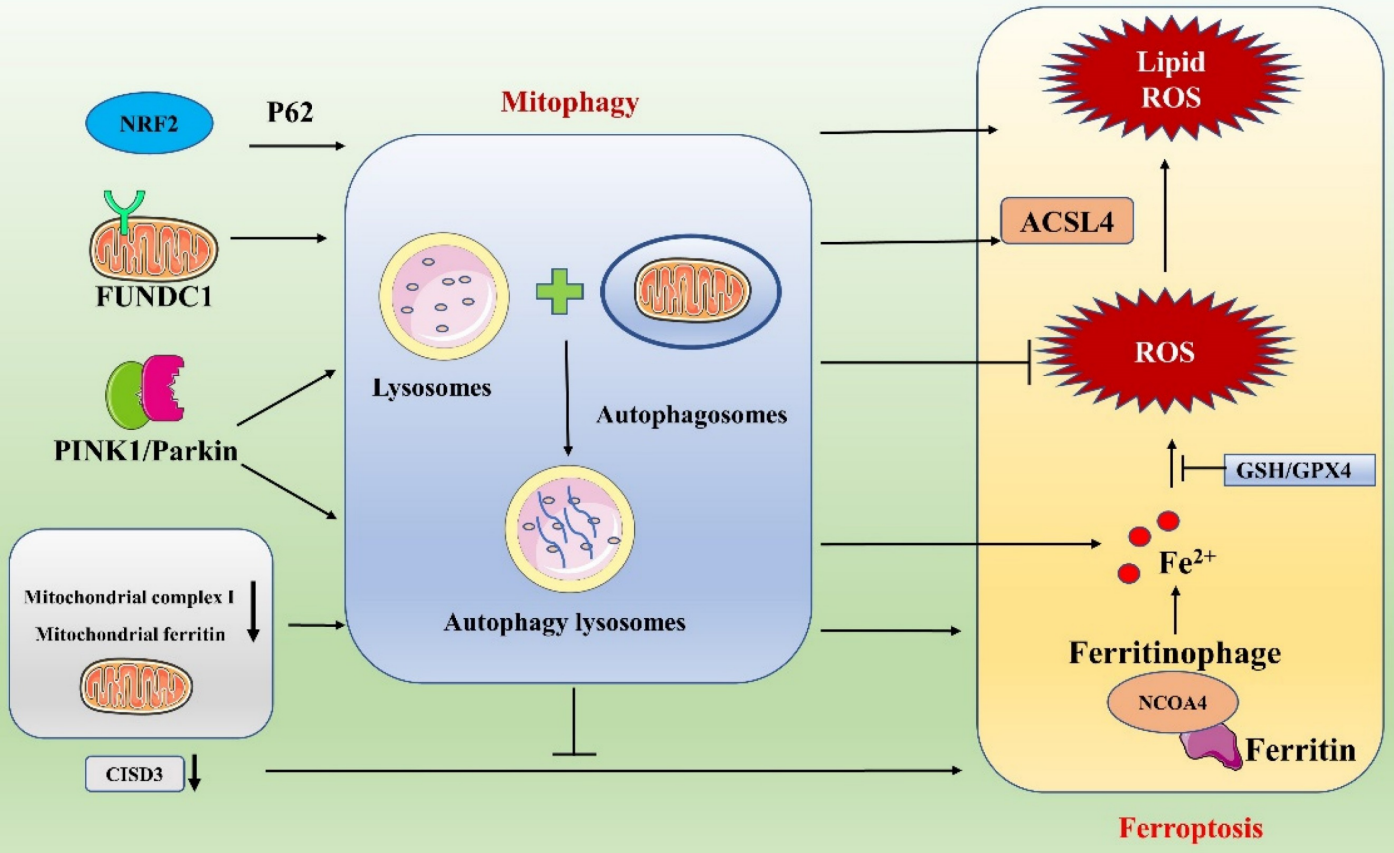
Link between ferroptosis and mitophagy.

As a type of selective autophagy, mitophagy plays a pivotal role in maintaining mitochondrial homeostasis by removing damaged mitochondria. Mitophagy is a mechanism for selective degradation of damaged mitochondria through autophagic flux and occurs via ubiquitin-dependent and ubiquitin-independent pathways 95. When cells are pathologically altered, damaged or dysfunctional mitochondria cause further damage to cells by producing large amounts of ROS and releasing proapoptotic factors. Therefore, timely removal of damaged mitochondria is essential for cellular homeostasis and viability. Ferroptosis may lead to cell damage, and mitophagy might play a protective role by suppressing the release of ROS from dysfunctional mitochondria **(Figure 7)** 96. Interestingly, mitophagy can either enhance or suppress ferroptosis. In response to mild stress or in the early stages of iron overload, mitophagy may sequester iron in mitophagosomes and thus reduce the amounts of source materials for ROS in ferroptosis 97. However, extensive mitophagy may ultimately provide additional iron, which amplifies lipid peroxidation and ferroptosis. Therefore, the degree of autophagic flux during mitophagy may vary, and the effects of mitophagy on ferroptosis may differ. As research has advanced, some inhibitors of ferroptosis (e.g., some antioxidants) have been shown to affect mitophagy; similarly, certain drugs related to the regulation of mitophagy also exert important effects on ferroptosis 96, 98. The synergistic effects of these drugs are significant in the treatment of diseases. For example, the compound WJ460, which targets the oncoprotein Myoferlin, synergistically promotes mitophagy and ROS accumulation in pancreatic cancer cells with the ferroptosis inducers erastin and RSL3, thereby leading to ferroptosis. In contrast, the mitochondrial inhibitor Mdivi1 and an iron chelator inhibit ROS production and restore cell proliferation 98. These findings suggest new research ideas for sensitivity studies with certain anticancer drugs.

In recent years, an increasing number of researchers have linked mitophagy with ferroptosis and have explored their associations and mechanisms of action in depth. FUNDC1, one of the receptor proteins that mediates mitophagy, has a key link with ferroptosis. The relationship between FUNDC1-mediated mitophagy and ferroptosis has been studied in detail in the context of cardiovascular disease by Peng and Pei et al 99, 100. In a study on paraquat toxicity to the heart, Peng et al. found that FUNDC1/JNK-mediated ferroptosis plays an important role in cardiac and mitochondrial damage induced by paraquat exposure: it prevented paraquat-induced mitophagy and ferroptosis when FUNDC1 was absent and effectively reduced cardiac injury 99. Pei et al. found that FUNDC1 deficiency caused metabolic and cardiac remodeling/contraction dysfunction in mice on a short-term high-fat diet. Specifically, FUNDC1 regulates ferroptosis via ACSL4 and influences the course of the disease 100. These findings support an interaction between FUNDC1 and ferroptosis and suggest that FUNDC1 may serve as a target for the diagnosis and treatment of cardiovascular disease. In addition, Granata et al. analyzed ferroptosis and mitophagy in the context of renal transplantation 96. They concluded that during I/R injury in kidney transplantation, oxidative stress is a key activator of several pathways, including the ferroptosis and mitophagy pathways. Ferroptosis may lead to renal damage, whereas mitophagy may play a protective role by reducing the release of ROS via elimination of dysfunctional mitochondria. A deeper understanding of these two pathways may lead to the identification of a new noninvasive biomarker for early detection of delayed graft function or to the development of clinically translatable pharmacological strategies. In our in-depth study, we found that ferroptosis and mitophagy are mediated through many interacting network nodes that can influence each other and play unique roles. For example, ferritinophagy plays a critical role in ferroptosis, and interactions between mitophagy and ferritinophagy pathways can promote ferroptosis 97. Fan et al. found that a major trophic sensor of glucose flux (protein O-GlcNAcylation) plays an important coordinating role in ferroptosis and mitophagy. Specifically, they found that protein O-GlcNAcylation regulates ferritin uptake and mitochondrial behavior, thereby controlling ferroptosis. When ferroptosis is induced, O-GlcNAc transferase is inactivated, leading to de-O-GlcNAcylation of ferritin, which activates NCOA4-mediated ferritinophagy and mitophagy; together, ferroptosis and ferritinophagy provide sources of unstable iron necessary for the Fenton reaction, leading to accelerated ROS production and lipid peroxidation 97. Moreover, simultaneous inhibition of ferritinophagy and mitophagy (e.g., double knockdown of NCOA4 and PINK1) almost completely blocks ferroptosis. Similarly, Singh et al. suggested that mitochondrial phagocytosis, ferritin phagocytosis, and lysosomal destabilization may play key roles in ferroptosis 101. In their study of diabetic retinopathy (DR), Singh et al. found that high levels of glucose induced TXNIP upregulation and that the associated redox stress caused mitochondrial dysfunction, mitophagy, ferritinophagy, and lysosomal destabilization. Then, unstable ions reacted with hydrogen peroxide to generate hydroxyl radicals and cause membrane phospholipid peroxidation due to reduced GSH levels and GPX4 activity, triggering ferroptosis.

Interestingly, abnormalities in certain mitochondrial molecules and functions are known to affect mitophagy and ferroptosis. For example, inhibition of mitochondrial complex I can trigger an increase in mitophagy-dependent ROS production, leading to ferroptosis in melanoma cells 102. Li et al. found that knockdown of the mitochondria-localized protein CISD3 significantly accelerates lipid peroxidation and exacerbates iron aggregation triggered by System Xc- suppression or cystine deprivation, which in turn promotes cellular ferroptosis. In contrast, activation of mitophagy alleviates CISD3 knockdown-induced ferroptosis by eliminating damaged mitochondria 103. Furthermore, maintenance of mitochondrial iron homeostasis plays an important role in the prevention of ferroptosis. Mitochondrial ferritin deficiency promotes ferroptosis mediated via mitophagy in osteoblasts of type 2 diabetic osteoporosis patients 104. In conclusion, a close association between mitochondrial autophagy and ferroptosis has been reported, and an in-depth exploration of the mechanisms involved in this association will provide new insights for future studies on certain diseases.

## Discussion

With a deepening understanding of ferroptosis, an increasing number of researchers have found that the maintenance of normal mitochondrial function plays an important role in ferroptosis **(Table 3)**^5^. Mitochondrial maintenance depends on a series of processes, such as mitochondrial fission, mitochondrial fusion and mitophagy, that are regulated by mitochondria themselves. Mitochondria undergo self-regulation through continuous fission and fusion, thereby maintaining the stability of mitochondria and cells. Through mitophagy, damaged mitochondria are eliminated from cells, thereby maintaining intracellular mitochondrial homeostasis. Mitophagy and mitochondrial fission/fusion are critical pathways that interact with and regulate one another to maintain mitochondrial homeostasis and normal cellular biological functions 105. In contrast, ferroptosis is an iron-dependent cell death modality induced by excessive ROS and lipid peroxide production. During the onset of ferroptosis, the main pathway of ROS production is in mitochondria. Dysregulation of mitochondrial function leads to an energy crisis, coordinates ferroptosis, and affects the cellular redox state. Thus, mitochondrial dynamics regulatory processes are an important means of maintaining mitochondrial quality control. Numerous mitochondria-regulated molecules, such as the mitochondrial fission genes DRP1 and FIS1, the mitochondrial fusion-related genes MFN1/2 and OPA1, and the mitophagy-related genes PINK1 and FUNDC1, are involved in ferroptosis. Changes in these genes profoundly affect mitochondrial function through different pathways, thus affecting intracellular oxidative and antioxidant systems and ultimately inhibiting or promoting ferroptosis. In addition, ferroptosis and mitochondrial dynamics are synergistically regulated, and an imbalance between the two disrupts intracellular homeostasis. For example, simultaneous inhibition of ferritinophagy and mitophagy (through double knockdown of NCOA4 and PINK1) almost blocks ferroptosis induced by O-GlcNAcylation 97. Mitochondria thus play an important role in the initiation of ferroptosis; for example, the ferroptosis agonist erastin directly targets the mitochondrial anion channel to induce ferroptosis 35. Given the evidence, the role of mitochondria in ferroptosis is unquestionable.

However, the specific mechanism of ferroptosis and mitochondrial dynamics regulation has not been fully explored, and the specific molecular network needs to be further constructed. Many issues remain to be addressed. (1) Interestingly, the relationship between ferroptosis and mitochondrial fission/fusion and mitophagy does not point to a single outcome; i.e., mitochondrial regulation alone can either promote or inhibit ferroptosis 96, 97. For example, in a study on mitochondrial fusion and ferroptosis, Li et al. found that an increased supply of ROS and fatty acids during mitochondrial fusion promotes ferroptosis 89; however, Shi et al. found that promoting mitochondrial fusion via treatment of disease with selenium attenuates oxidative stress and inhibits ferroptosis 90. Similarly, a study on ferroptosis and mitophagy revealed that normal mitophagy contributes to ferroptosis inhibition, while excessive mitophagy pathway activation promotes ferroptosis. These findings suggest that intracellular mitochondrial fission and fusion and mitophagy are dynamic regulatory processes that may exert opposite effects on ferroptosis depending on the degree of their regulatory impact. Therefore, strategies to promote a regulatory balance in mitochondria are becoming important directions for future research. (2) A complex network of genes regulates ferroptosis, mitochondrial fission, mitochondrial fusion and mitophagy. For example, NRF2 is a key hub gene in the regulation of ferroptosis and mitochondrial dynamics, and it has multiple regulatory effects on mitochondrial biogenesis, mitochondrial fission and fusion, and mitophagy 33, 34. Precise identification of intersecting nodes in the NRF2 interaction network is key to understanding its role in the regulation of these biological processes. There are still many key genes whose roles remain undetermined; these need to be explored. (3) The dynamic regulation of ferroptosis and mitochondria has a significant role in the development and treatment of tumors 85, 89, 98. Coordinated bidirectional regulation of ferroptosis and mitochondria can enhance tumor cell death and thus induce therapeutic effects. This may provide research possibilities for the development of new antitumor drugs and for combination treatments. Studying the interaction networks between the two and finding their focused intersection targets will provide important references to direct the development and application of oncology drugs. However, finding precise regulatory targets is difficult. (4) There is a close link between ferroptosis and mitochondrial biogenesis. For example, NRF2, a key factor in ferroptosis, can affect mitochondrial biogenesis by regulating the expression of PGC-1α, NResF1, NResF2, TFAM and mitochondrial genes 32, 34. In addition, NRF2 has been shown to represent an important link between mitochondrial fission, mitochondrial fusion and mitophagy. Thus, there is a complex regulatory network among the three processes, which interact with and influence each other. However, the exact regulatory network is still unclear.

Although many questions remain to be addressed, mitochondrial fission, mitochondrial fusion and mitophagy clearly exert significant effects on ferroptosis. Studying these biological mechanisms may lead to new ideas for the prevention and treatment of diseases in the future.

## Summary

In conclusion, mitochondrial fission and fusion and mitophagy maintain mitochondrial homeostasis and have important effects on ferroptosis. A deeper understanding of the relationships among these regulatory processes may lead to the development of new preventive and therapeutic strategies for future disease research.

## Figures and Tables

**Table 1:** Mechanisms underlying ferroptosis

Group	Pathways	Key targets	Mechanisms
The exogenous pathway	Inhibition of system Xc- induced ferroptosis	system Xc-, GPX4, p53	system Xc-/GSH/GPX4/ROS/Lipid ROS
			p53/ system Xc-
The endogenous pathway	Lipid metabolism-induced ferroptosis	ACSL4, LPCAT3, LOX	PUFAs/ ACSL4 and LPCAT3/PUFA-PE/LOX/ Lipid ROS
	Iron metabolism-regulated ferroptosis	TRF1, Ferritin, LIP, Fpn, HO-1, HSPB1, NCOA4, NRF2	Fe^3+^/ TFR1/ STEAP3/ Fe^2+^/ DMT1 or ZIP8/14/LIP/ROS
			Fe^2+^/ Fpn/ Fe^3+^
			HO-1/ Fe^2+^/LIP
			HSPB1/ TRF1
			NCOA4/ Ferritin/ Fe^2+^/LIP
			NRF2/ TRF1, Ferritin, HO-1, Fpn/ Fe^2+^/LIP
	Ferroptosis with mitochondrial involvement	GPX4, DHODH, NRF2, VDACs	ATP/AMPK/acetyl-CoA carboxylase
			TCA cycle/ electron transport and fatty acid biosynthesis
			GPX4/ CoQ/CoQH2
			DHODH/ CoQ/CoQH2
			NRF2/ mitochondrial biogenesis
			NRF2/ mitophagy (P62-dependent, PINK1/Parkin-independent)
			NRF2/ proteasomal genes/ mitochondrial fission
			NRF2/ MFN2/ mitochondrial fusion
			Erastin/VDACs/ Lipid ROS
	Others	FSP1, GPX4, P53	FSP1-COQ10-NAD(P)H
			P53/STAT1/ALOX15/Lipid ROS
			Sulfur transfer pathway

ACSL4 Acyl-CoA synthetase long-chain family member 4; AMPK: AMP-activated protein kinase; DHODH: dihydroorotate dehydrogenase; DMT1: divalent metal transporter 1; GPX4: glutathione peroxidase 4; GSH: glutathione; HO-1: Heme oxygenase-1; HSPB1: heat shock protein B1; LIP: unstable iron pool; LOX: lipoxygenase; LPCAT3: lysophosphatidylcholine acyltransferase 3; MFN2: mitochondrial fusion protein 2; NCOA4: nuclear receptor coactivator 4; NRF2: nuclear factor red lineage 2 related factor 2; PINK1: PTEN-induced kinase 1; PUFAs: polyunsaturated fatty acids; ROS: reactive oxygen species; STEAP3: six-transmembrane epithelial antigen of prostate 3; TFR1: transferrin receptor protein 1; VDACs : mitochondrial voltage-dependent anion channels;ZIP8/14 zinc-iron regulatory protein family 8/14

**Table 2:** Dynamic regulation of mitochondria

	Pathways		Key genes	Mechanisms	Roles
**Mitochondrial fission**	DRP1-mediated pathway		DRP1, Mff, Fis1, MiD49, MiD51	The recruited DRP1 binds to the protein receptor (Mff, Fis1, MiD49 and MiD51), which breaks the inner and MOM, leading to mitochondrial fission.	Splitting of damaged mitochondria into two types of mitochondria with normal and abnormal function.
**Mitochondrial fusion**	MFN1, MFN2 and OPA1-mediated pathways		MFN1, MFN2, OPA1	The fusion of the MOM is mainly mediated by MFN1 and MFN2, while the fusion of the MIM is mediated by OPA1.	Maintains mitochondrial homeostasis by fusing damaged mitochondria with normal mitochondria to perform relatively normal functions.
**Mitophagy**	PINK1/Parkin pathways		PINK1/Parkin	PINK1 aggregates at the MOM and recruits and activates Parkin, which in turn activates Parkin to polyubiquitinate a variety of mitochondrial protein substrates. Ultimately, mitophagy is induced by the action of LC3.	Mitophagy can wrap and remove damaged mitochondria from the cell through the autophagic pathway, thereby maintaining intracellular mitochondrial homeostasis.
	PINK1/Parkin-independent pathways		OPTN, NDP52, ULK1, DFCP1, WIPI1, synphilin-1	PINK1 directly recruits OPTN and NDP52 to damaged mitochondria and subsequently recruits and activates ULK1, DFCP1 and WIPI1, which in turn induce mitophagy. PINK1-synphilin-1 complex induces mitophagy by recruiting SIAH-1 to accelerate the ubiquitination of proteins in damaged mitochondria.	
	Receptor-mediated pathways	Receptors in the MOM	NIX/BNIP3L, BNIP3, FUNDC1, NLRX1, FKBP8, Bcl2L13	The receptors located in the MOM all contain a binding site for the LC3 receptor, which can directly bind LC3 to induce mitophagy.	
		Receptors in the MIM	PHB2 and CL	Mitochondrial damage leads to the exposure of PHB2, which in turn binds to LC3; moreover, PHB2 induces mitophagy by stabilizing PINK1 and promoting PRKN/Parkin recruitment, protein ubiquitination and OPTN recruitment to mitochondria. CL transfer from MIM to MOM directly interacts with LC3 to induce mitophagy	

DRP1: dynamin-related protein 1; Fis1: mitochondrial fission protein 1; Mff: mitochondrial fission factor; MFN1: mitochondrial fusion protein 1; MFN2: mitochondrial fusion protein 2; MiD49: mitochondrial dynamics protein 49; MiD51: mitochondrial dynamics protein 51; MIM: mitochondrial inner membrane; MOM: mitochondrial outer membrane; OPA1: optic atrophy 1 protein; OPTN: Optineurin; PINK1: PTEN-induced kinase 1; ULK1: unc-51 like kinase 1; WIPI1: WD repeat domain phosphoinositide interacting 1

**Table 3:** Ferroptosis and Mitochondrial Fission, Fusion, Mitophagy

Group	Mitochondrion	Ferroptosis	Mechanism	Related articles	Ref.
Ferroptosis and mitochondrial fission	DRP1 Fis1 Mff	NRF2, ROS, lipid peroxidation	Vitamin D3/ DRP1, Fis1/ROS/ lipid peroxidation	Vitamin D3 attenuates cisplatin-induced intestinal injury by inhibiting ferroptosis, oxidative stress, and ROS-mediated excessive mitochondrial fission.	84
			Erastin/ROS/DRP1, Fis1, MFF/ mitophagy (PINK1, Parkin)	Ferroptosis inducer erastin sensitizes NSCLC cells to celastrol through activation of the ROS-mitochondrial fission-mitophagy axis.	85
			NRF2/ proteasomal genes /DRP1	The Keap1-Nrf2 Stress Response Pathway Promotes Mitochondrial Hyperfusion Through Degradation of the Mitochondrial Fission Protein Drp1	83
			CA9/ Mitochondrial fission	Carbonic anhydrase 9 confers resistance to ferroptosis/apoptosis in malignant mesothelioma under hypoxia.	86
			Mitochondrial fission/p53/SLC7A11/GPX4	SHARPIN promotes cell proliferation of cholangiocarcinoma and inhibits ferroptosis via p53/SLC7A11/GPX4 signaling.	87
Ferroptosis and mitochondrial fusion	MFN1 MFN2 OPA1	NRF2, ROS, lipid peroxidation	NRF2/MFN2	Sulforaphane enriched transcriptome of lung mitochondrial energy metabolism and provided pulmonary injury protection via Nrf2 in mice	88
			Erastin /STING1/ MFN1/2/ROS/ lipid peroxidation/ferroptosis	STING1 Promotes Ferroptosis Through MFN1/2-Dependent Mitochondrial Fusion.	89
			Selenium/ MFN1/ROS/ferroptosis	Selenium Alleviates Cerebral Ischemia/Reperfusion Injury by Regulating Oxidative Stress, Mitochondrial Fusion and Ferroptosis.	90
			QSYQ/ MFN2, OPA1	QiShenYiQi dripping pill alleviates myocardial ischemia-induced ferroptosis via improving mitochondrial dynamical homeostasis and biogenesis.	91
Ferroptosis and mitophagy	PINK1 Parkin FUNDC1	System Xc-, GPX4, ACSL4, NRF2, ROS, Fe^2+^, NCOA4, lipid peroxidation	Mitophagy/dysfunctional mitochondria/ROS/ferroptosis	Oxidative Stress and Ischemia/Reperfusion Injury in Kidney Transplantation: Focus on Ferroptosis, Mitophagy and New Antioxidants.	96
			Myoferlin/ mitophagy/ System Xc-/GPX4/ROS	Myoferlin targeting triggers mitophagy and primes ferroptosis in pancreatic cancer cells.	98
			Dynamic O-GlcNAcylation/ ferritinophagy (NCOA4), mitophagy/ Fe^2+^/ROS/ lipid peroxidation/ ferroptosis	Dynamic O-GlcNAcylation coordinates ferritinophagy and mitophagy to activate ferroptosis.	97
			NRF2/ mitophagy (P62-dependent, PINK1/Parkin-independent)	PMI: a DeltaPsim independent pharmacological regulator of mitophagy.	33
			High Glucose/TXNIP/ ferritinophagy (NCOA4), mitophagy/ Fe^2+^, GSH, GPX4/ROS/ lipid peroxidation/ ferroptosis	Mitophagy, Ferritinophagy and Ferroptosis in Retinal Pigment Epithelial Cells Under High Glucose Conditions: Implications for Diabetic Retinopathy and Age-Related Retinal Diseases.	101
			FUNDC1/JNK/ lipid peroxidation/ferroptosis	Ablation of FUNDC1-dependent mitophagy renders myocardium resistant to paraquat-induced ferroptosis and contractile dysfunction.	99
			FUNDC1/ACSL4	FUNDC1 insufficiency sensitizes high fat diet intake-induced cardiac remodeling and contractile anomaly through ACSL4-mediated ferroptosis.	100
			Erastin/ROS/DRP1, Fis1, MFF/ mitophagy (PINK1/Parkin)	Ferroptosis inducer erastin sensitizes NSCLC cells to celastrol through activation of the ROS-mitochondrial fission-mitophagy axis.	85
			Mitochondrial complex I/ mitophagy (PINK1)/ROS/ferropsosis	Mitochondrial complex I inhibition triggers a mitophagy-dependent ROS increase leading to necroptosis and ferroptosis in melanoma cells.	102
			Mitophagy/CISD3/ lipid peroxidation	CISD3 inhibition drives cystine-deprivation induced ferroptosis. Cell Death Dis	103
			Mitochondrial Ferritin Deficiency / Fe^2+^/ROS / mitophagy (PINK1/Parkin)/ferroptosis	Mitochondrial Ferritin Deficiency Promotes Osteoblastic Ferroptosis Via Mitophagy in Type 2 Diabetic Osteoporosis.	104

ACSL4 Acyl-CoA synthetase long-chain family member 4; DRP1: dynamin-related protein 1; Fis1: mitochondrial fission protein 1; GPX4: glutathione peroxidase 4; GSH: glutathione; HIF-1 hypoxia-inducible factor 1; Mff: mitochondrial fission factor; MFN1: mitochondrial fusion protein 1; MFN2: mitochondrial fusion protein 2; NCOA4: nuclear receptor coactivator 4; NRF2: nuclear factor red lineage 2 related factor 2; NSCLC: non-small cell lung cancer; OPA1: optic atrophy 1 protein; PINK1: PTEN-induced kinase 1; PUFAs: polyunsaturated fatty acids; ROS: reactive oxygen species

## Abbreviations

AA: arachidonic acid
ACSL4: acyl-CoA synthetase long-chain family member 4
AMPK: AMP-activated protein kinase
DFCP1: double FYVE-containing protein 1
DHODH: dihydroorotate dehydrogenase
DMT1: divalent metal transporter 1
DNM1L: dynamin-1-like protein
DRP1: dynamin-related protein 1
Fis1: mitochondrial fission protein 1
GPX4: glutathione peroxidase 4
GSH: glutathione
HIF-1: hypoxia-inducible factor 1
HO-1: heme oxygenase-1
HSPB1: heat shock protein B1
IMS: intermembrane space
LIP: labile iron pool
LOX: lipoxygenase
LPCAT3: lysophosphatidylcholine acyltransferase 3
MARCH5: membrane-associated ring finger protein 5
MCAO: middle cerebral artery occlusion
Mff: mitochondrial fission factor
MFN1: mitochondrial fusion protein 1
MFN2: mitochondrial fusion protein 2
MiD49: mitochondrial dynamics protein 49
MiD51: mitochondrial dynamics protein 51
MIM: mitochondrial inner membrane
MOM: mitochondrial outer membrane
MTS: mitochondrial targeting sequence
NCOA4: nuclear receptor coactivator 4
NLR: NOD-like receptor
NRF2: nuclear factor red lineage 2 related factor 2
NSCLC: non-small cell lung cancer
OPA1: optic atrophy 1 protein
OPTN: Optineurin
PE: phosphatidylethanolamine
PGC-1α: proliferator-activated receptor gamma coactivator 1α
PINK1: PTEN-induced kinase 1
PUFAs: polyunsaturated fatty acids
ROS: reactive oxygen species
STEAP3: six-transmembrane epithelial antigen of prostate 3
TFR1: transferrin receptor protein 1
ULK1: unc-51 like kinase 1
WIPI1: WD repeat domain phosphoinositide interacting 1
ZIP8/14: zinc-iron regulatory protein family 8/14
